# Supplementary material for: COZOID: contact zone identifier for visual analysis of protein-protein interactions
Source: BMC Bioinformatics. 2018 Apr 6;19:125. doi: 10.1186/s12859-018-2113-6 (PMC5889581; doi:10.1186/s12859-018-2113-6)
Supplement: Supplementary file 2 — Software build. Executable binary file of the software tool. (ZIP 118,784 kb) [file 12859_2018_2113_MOESM2_ESM.zip › cozoid/java/docs/beansbinding-1.2.1-doc.zip!/org/jdesktop/el/impl/parser/TokenMgrError.html]

TokenMgrError (Beans Binding)


---


|  |  |  |
| --- | --- | --- |
| **Overview** \| **Package** \| **Class** \| **Use** \| **Tree** \| **Deprecated** \| **Index** \| **Help** | |  |
| **PREV CLASS**   NEXT CLASS | **FRAMES**    **NO FRAMES**     **All Classes** |  |
| SUMMARY: NESTED \| FIELD \| CONSTR \| METHOD | DETAIL: FIELD \| CONSTR \| METHOD |  |


---


## org.jdesktop.el.impl.parser Class TokenMgrError

```
java.lang.Object
  java.lang.Throwable
      java.lang.Error
          org.jdesktop.el.impl.parser.TokenMgrError
```

**All Implemented Interfaces:**: java.io.Serializable

---

``` public class TokenMgrError extends java.lang.Error ```

**See Also:**: Serialized Form

---

**Constructor Summary**

`TokenMgrError()`

`TokenMgrError(boolean EOFSeen,
int lexState,
int errorLine,
int errorColumn,
java.lang.String errorAfter,
char curChar,
int reason)`

`TokenMgrError(java.lang.String message,
int reason)`


| **Method Summary** | |
| --- | --- |
| `protected static java.lang.String` | `addEscapes(java.lang.String str)`             Replaces unprintable characters by their espaced (or unicode escaped) equivalents in the given string |
| `java.lang.String` | `getMessage()`             You can also modify the body of this method to customize your error messages. |
| `protected static java.lang.String` | `LexicalError(boolean EOFSeen, int lexState, int errorLine, int errorColumn, java.lang.String errorAfter, char curChar)`             Returns a detailed message for the Error when it is thrown by the token manager to indicate a lexical error. |

**Methods inherited from class java.lang.Throwable**

`fillInStackTrace, getCause, getLocalizedMessage, getStackTrace, initCause, printStackTrace, printStackTrace, printStackTrace, setStackTrace, toString`

**Methods inherited from class java.lang.Object**

`clone, equals, finalize, getClass, hashCode, notify, notifyAll, wait, wait, wait`

**Constructor Detail**

### TokenMgrError

```
public TokenMgrError()
```

---


### TokenMgrError

```
public TokenMgrError(java.lang.String message,
                     int reason)
```

---


### TokenMgrError

```
public TokenMgrError(boolean EOFSeen,
                     int lexState,
                     int errorLine,
                     int errorColumn,
                     java.lang.String errorAfter,
                     char curChar,
                     int reason)
```


**Method Detail**

### addEscapes

```
protected static final java.lang.String addEscapes(java.lang.String str)
```

:   Replaces unprintable characters by their espaced (or unicode escaped)
    equivalents in the given string

---


### LexicalError

```
protected static java.lang.String LexicalError(boolean EOFSeen,
                                               int lexState,
                                               int errorLine,
                                               int errorColumn,
                                               java.lang.String errorAfter,
                                               char curChar)
```

:   Returns a detailed message for the Error when it is thrown by the
    token manager to indicate a lexical error.
    Parameters :
    EOFSeen : indicates if EOF caused the lexicl error
    curLexState : lexical state in which this error occured
    errorLine : line number when the error occured
    errorColumn : column number when the error occured
    errorAfter : prefix that was seen before this error occured
    curchar : the offending character
    Note: You can customize the lexical error message by modifying this method.

---


### getMessage

```
public java.lang.String getMessage()
```

:   You can also modify the body of this method to customize your error messages.
    For example, cases like LOOP\_DETECTED and INVALID\_LEXICAL\_STATE are not
    of end-users concern, so you can return something like :
    "Internal Error : Please file a bug report .... "
    from this method for such cases in the release version of your parser.

    :   **Overrides:**: `getMessage` in class `java.lang.Throwable`


---


|  |  |  |
| --- | --- | --- |
| **Overview** \| **Package** \| **Class** \| **Use** \| **Tree** \| **Deprecated** \| **Index** \| **Help** | |  |
| **PREV CLASS**   NEXT CLASS | **FRAMES**    **NO FRAMES**     **All Classes** |  |
| SUMMARY: NESTED \| FIELD \| CONSTR \| METHOD | DETAIL: FIELD \| CONSTR \| METHOD |  |


---
